# Barriers and enablers of breast cancer screening among women in East Africa: a systematic review

**DOI:** 10.1186/s12889-023-16831-0

**Published:** 2023-10-04

**Authors:** Faraja Mussa Magwesela, Doreen Ombeni Msemakweli, David Fearon

**Affiliations:** 1 Arusha Lutheran Medical Centre, P.O. Box 17047, Arusha, Tanzania; 2 College of Medicine and Veterinary Medicine, University of Edinburgh, Old College, South Bridge, Edinburgh, EH8 9YL UK

**Keywords:** Breast cancer, Breast cancer screening, East Africa, Barriers, Enablers

## Abstract

**Background:**

Breast cancer is among the most common cancers globally with a projected increase in incidence and mortality in low- and middle-income countries. The majority of the patients in East Africa present with advanced disease contributing to poor disease outcomes. Breast cancer screening enables earlier detection of the disease and therefore reduces the poor outcomes associated with the disease. This study aims to identify and synthesize the reported barriers and enablers of breast cancer screening among East African women.

**Methods:**

Medline, Embase, SCOPUS, and Cochrane library were searched for articles published on the subject from start to March 2022 using PRISMA guidelines. Also, forward citation, manual search of references and searching of relevant journals were done. A thematic synthesis was carried out on the “results/findings” sections of the identified qualitative papers followed by a multi-source synthesis with quantitative findings.

**Results:**

Of 4560 records identified, 51 were included in the review (5 qualitative and 46 quantitative), representing 33,523 women. Thematic synthesis identified two major themes – “Should I participate in breast cancer screening?” and “Is breast cancer screening worth it?”. Knowledge of breast cancer and breast cancer screening among women was identified as the most influencing factor.

**Conclusion:**

This review provides a rich description of factors influencing uptake of breast cancer screening among East African women. Findings from this review suggest that improving knowledge and awareness among both the public and providers may be the most effective strategy to improve breast cancer screening in Eastern Africa.

**Supplementary Information:**

The online version contains supplementary material available at 10.1186/s12889-023-16831-0.

## Background

Breast cancer (BC) is among the most commonly diagnosed malignancy globally [1, 2]. According to GLOBOCAN 2020, BC accounted for the majority of new cancer cases diagnosed globally (2.3million people diagnosed) and contributed to 6.9% of cancer-related deaths (ranked fifth after lung (18%), colorectal (9.4%), liver (8.3%) and stomach cancers (7.7%)) [3]. It is projected that its incidence will continue to increase, mostly in low- and middle-income countries (LMICs) as a result of population aging and increased adoption of high-risk lifestyles [2, 4].

The distribution of BC varies between countries, the incidence being higher in high-income countries (HICs) than LMICs though most of the deaths related to BC occur in LMICs [3]. LMICs face an unproportionally high burden of disease compared to HICs due to the majority of the patients presenting with advanced disease necessitating complex treatment options which are often absent in these areas [5, 6].

Sub-Saharan Africa has experienced rapid increase in BC incidence over the last 20-30 years, and it has the highest mortality rates in the world [3, 7]. This has been attributed to delayed patient presentation and weak health infrastructure [5, 7]. Delayed presentations have been attributed to – inefficient screening services, insufficient healthcare infrastructure, unavailability and high cost of cancer services, and low patient awareness about the disease [8–12]. In East Africa (EA), the incidence of BC in 2020 was estimated at 33 per 100,000 person-years, whereas the mortality was estimated at 17.9 per 100,000 person-years (compared 50.4 vs 15.7 in Southern Africa, 41.5 vs 22.3 in Western Africa and 32.7 vs 18 in Central Africa, incidence vs mortality rate per 100,000 persons) [3].

The disease stage at the time of diagnosis is a significant determinant of survival. Early-stage disease is associated with better survival than advanced disease [7, 13]. Due to most patients in Sub-Saharan Africa presenting with advanced disease, it is imperative to improve programs (such as breast cancer screening) that will increase the early detection of disease. Efforts to promote screening, followed by early and appropriate treatment are essential components to improving survival. Whereas screening programs focus on asymptomatic patients, early detection programs focus on patients with early symptoms of disease, both being essential in early cancer diagnosis.

Breast cancer screening (BCS) methods commonly used in East Africa (EA) are – self-breast examination (BSE), clinical breast examination (CBE), ultrasonography and mammography [14]. Mammography is currently the gold standard of BCS [14]. Most guidelines recommend annual or biennial mammographic screening between 40 and 74 years for average-risk populations and annual mammography or annual magnetic resonance imaging starting from a younger age for high-risk populations. In resource-limited settings like EA, population-based mammography screening has not been considered to be cost-effective and other cost-effective methods (CBE and BSE) have to be used [3]. Other methods of BCS that are available though not commonly used in East Africa are; magnetic resonance imaging, molecular imaging and genetic testing [14].

Primary studies from EA have reported low uptake of BCS services, particularly mammography [8–12]. Several factors proposed as influencing screening uptake in other Sub-Saharan countries include – knowledge about BC and BCS, socio-cultural factors, economic factors, perception and attitude toward BC and BCS, provider factors and other related factors [1, 15–17]. We have identified no study that has systematically gathered evidence for factors influencing breast screening uptake in the EA region. East African region has been defined to include the countries Burundi, Comoros, Djibouti, Eritrea, Ethiopia, Kenya, Seychelles, Somalia, South Sudan, Sudan, Rwanda, Tanzania, and Uganda [18, 19].

In this study, we aimed to systematically review the published literature on the status of breast cancer screening in East Africa by examining the factors associated with uptake of the various methods used for BCS. We targeted Eastern Africa as it was shown that the cumulative risk of dying from cancer from women in 2020 according to GLOBOCAN was higher in Eastern Africa (11%) compared to other regions of the world [3]. results from this study may help policymakers and other stakeholders to identify gaps in breast cancer management and device pathways to improve early disease detection and reduce adverse outcomes.

## Methods

This review followed the Preferred Reporting Items for Systematic Reviews and Meta-Analyses (PRISMA) guidelines [20].

### Search strategy

A comprehensive electronic database literature search was conducted in March 2022 using Cochrane Library, MEDLINE, EMBASE and SCOPUS. To complement the database search, forward citation tracking and examination of reference lists of relevant studies were conducted. Finally, hand searching for articles in the following libraries was undertaken – African Journals Online, DiscoverEd, and Pan-African Medical Journal.

Population, intervention, comparison outcomes, timing and study type (PICOTS) approach was used to generate groups of medical subject headings (MeSH) terms and keywords. (See Table 1 for search terms and Additional file 1 for full MEDLINE search):

- Population – women residing in East African countries (as defined in the background).
- Intervention – any breast cancer screening method used.
- Comparison – not applicable
- Outcomes – influencers (barriers and facilitators) of breast cancer screening uptake.
- Timing – from start to March 2022 (included studies ranged from 2010 to 2022)
- Study type – quantitative studies, qualitative studies and primary mixed methods studies published in peer-reviewed journals.

**Table 1:** Search terms used

Condition		Location	Experience
(Breast OR Mammary)	(Cancer OR Tumour OR Tumor OR Malignancy OR Neoplasm)	Burundi, Comoros, Djibouti, Eritrea, Ethiopia, Kenya, Seychelles, Somalia, South Sudan, Sudan, Rwanda, Tanzania, and Uganda	Screening, Early Diagnosis, Early detection of cancer, barrier, Perception, Social Perception, opinion, Attitude to Health, attitude, social value, social norm, Culture, belief, understanding, language, communication, fear, Mistrust, trust, culture, religion, Knowledge, Embarrassment, fatalism, fatalistic, income, socioeconomic, deprivation, education, poor, poverty

To maximize retrieval of all relevant articles to give a complete picture of factors influencing BCS, the year of publication limitation was not imposed. Boolean operators “OR”, and “AND”, were used to include, and restrict search terms.

### Selection criteria

The inclusion criteria were as follows.

1. Population – studies conducted among women in Burundi, Comoros, Djibouti, Eritrea, Ethiopia, Kenya, Seychelles, Somalia, South Sudan, Sudan, Rwanda, Tanzania, or Uganda, regardless of race or ethnicity.
2. Intervention – studies reporting the use of any method of BCS.
3. Outcomes – studies reporting factors associated with uptake of BCS.
4. Study design – quantitative studies, qualitative studies and primary mixed methods studies published in peer-reviewed journals.
5. Studies reported in English.

### Exclusion criteria

1. Studies reporting BCS that failed to indicate factors related to the use/non-use of screening methods.
2. Studies where barriers/facilitators are not related to BCS.
3. Studies among women from East Africa residing in non-EA countries
4. Studies published in languages other than English
5. Grey literature, reviews, editorial, letter, book chapters, as well as abstracts with no full text were excluded.

### Study selection

All articles were retrieved through the electronic search process and entered into an EndNote bibliographic database. All retrieved studies had their titles and abstracts screened to assess for eligibility after duplicates were removed. Full-text articles were retrieved if eligibility was met and for those in doubt.

### Quality assessment

Qualitative studies were qualified using the Critical Appraisal Skills Programme (CASP) quality assessment tool (http//www.casp-uk.net). Quantitative studies were assessed using JBI’s cross-sectional critical appraisal tool (jbi. global/critical-appraisal-tools) (see Additional file 2 for criteria used for quality assessment). For both tools, each criterion was given a score from 0–2 based on the author’s judgement. These were then summed and an assessment of the overall quality of a study was ranked as “good”, “fair”, and “poor”. The quality score for quantitative studies ranged from 0–40 (0–20 = poor, 21–30 = fair, 21–40 = high). The quality score for qualitative studies ranged from 0–36 (0–18 = poor, 19–28 = fair, 29–36 = good). No studies were excluded as a result of the quality assessment, rather, the quality assessment contributed to the confidence of each finding.

### Data extraction and synthesis

Key data from each of the included papers were extracted using a template. Extracted data included the name of the first author; year of publication; country of study; study aims, design, setting, and demographics; data collection methods; sampling technique and sample size; BCS method investigated; and factors related to uptake of screening method(s).

Data extraction, analysis and synthesis for qualitative studies followed Thomas and Harden’s (2008) thematic synthesis approach [21]. All data found in the “findings” and/or “results” sections of both the abstracts and the main texts, including quotations from the study participants were exported verbatim into N-Vivo (N Vivo Qualitative Research Data Analysis Software QSR International Pty Ltd. 2020). Extracts were read and re-read followed by coding line by line. The codes were then grouped into clusters and finally into themes. Identified themes were examined for interconnectedness with included quantitative studies and the findings from the quantitative papers were absorbed within the themes using multi-source synthesis method [22]. Multi-source synthesis method was used as it offers a step by step approach to synthesis of data from multiple sources (both qualitative and quantitative) to reach a broader conclusion.

Narrative synthesis for quantitative studies following Popay et al., (2006), guidance for the conduct of narrative synthesis for systematic reviews [23], is provided in Additional file 3.

## Results

### Study characteristics

**Fig. 1:**
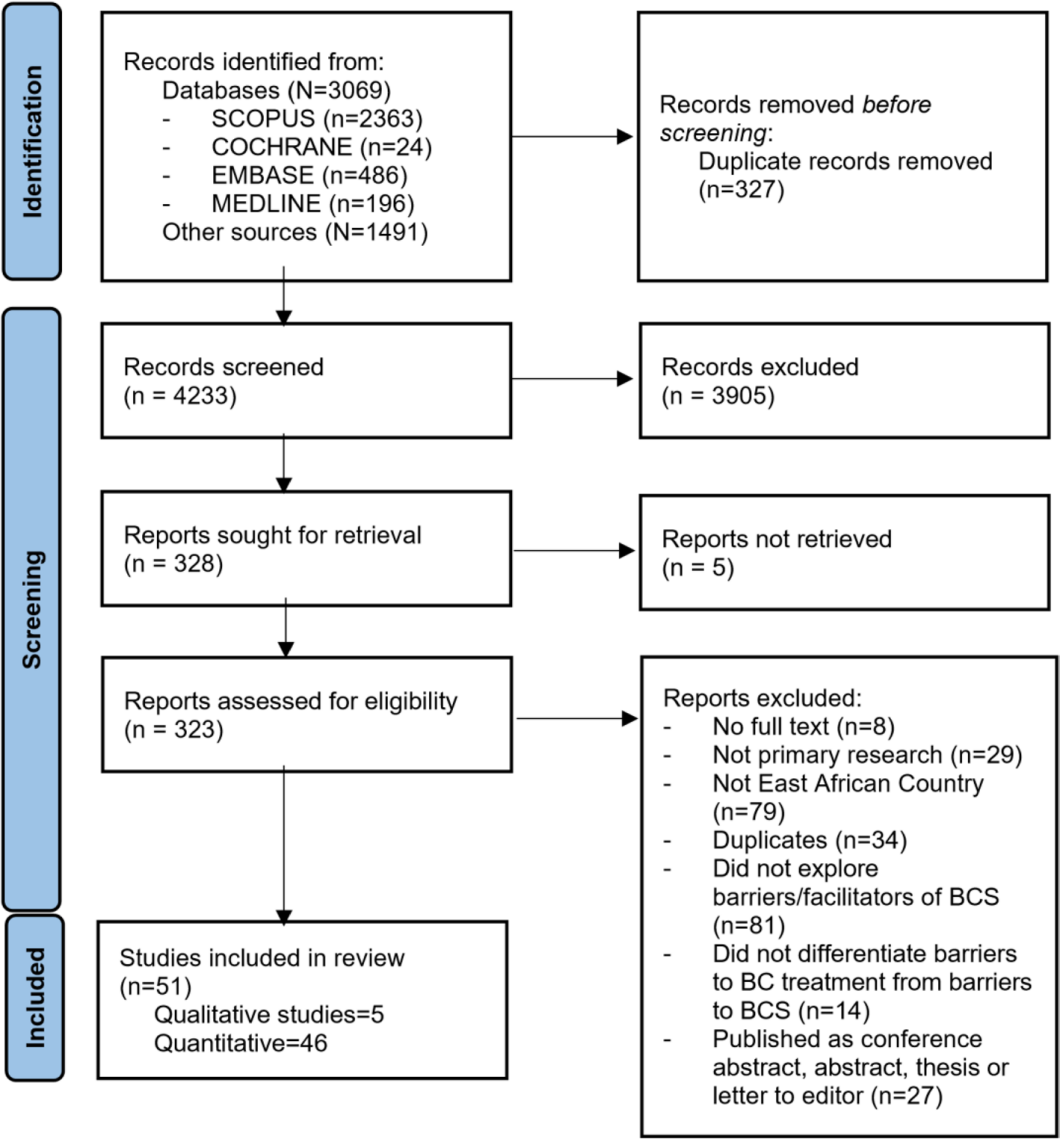
PRISMA framework for systematic review

The initial search yielded 4560 studies, out of which 51 were selected for inclusion in the study (see PRISMA flowchart in Fig. 1). Of the 51 included studies, five employed qualitative (Ethiopia = 2, Kenya = 2, Uganda = 1), and 46 quantitative methods of data collection (Eritrea = 1, Ethiopia = 33, Kenya = 4, Tanzania = 2, Uganda = 6). These represented data from 33,523 participants (22 breast cancer patients and 33,501 asymptomatic women) from five countries – Eritrea, Ethiopia, Kenya, Tanzania, and Uganda. The study characteristics are shown in Tables 2 and 3.

**Table 2:** Characteristics of included qualitative studies

First author	Year	Country	Study design	Study focus	Sample size	Study population	BCS method assessed	Factors identified	Overall quality
Agide	2019	Ethiopia	Focus group discussions and in-depth interviews	Explore perception about breast screening	64 CW and 9 HCWs *n* = 73	Reproductive age women	BSE, CBE and RS	Low awareness Cost of the services	Fair
Getachew	2020	Ethiopia	In-depth interview	Identify the perceived barriers to early diagnosis of BC	12 BC patients and 13 HCWs *n* = 25	BC patients and BC health providers from six public health hospitals in rural and urban south and southwestern Ethiopia	BSE, CBE and RS	Lack of knowledge and awareness Fear of losing a breast if cancer was diagnosed Lack of social and financial support to seek care Long distance to health facilities High cost of services Lack of screening tests in local facilities Long waiting time for screening tests	Good
Ilaboya	2017	Uganda	Semi-structured interviews, key informant interviews and focus group discussions	Perceived barriers to early detection of BC	*n* = 24	Women and community health workers in Wakiso District	BSE, CBE and RS	Lack of knowledge Fearful of BC diagnosis/prognosis/treatment Poor health-seeking behaviour Poverty, Focus on CDs Long waiting times No training of CHWs on NCDs Access to cancer services at the primary health care level	Good
Kisiagani	2018	Kenya	Focus group discussions and key informant interviews	Evaluate the knowledge, attitude and health-seeking behaviour towards BC and its screening	*n* = 72	Urban and rural women of Kakamega county	BSE, CBE and RS	Being married (Urban) Fatalistic views towards being diagnosed with BC lack of information on BC and its screening (Rural) Low-income status Not enough staff to perform CBE Emphasis on CDs, Long distance to the health facility	Fair
Muthoni	2010	Kenya	Focus group discussions	Knowledge, attitudes, and behaviours concerning BC and its early detection measures	*n* = 80	Urban and rural women in Machakos and Kiambu districts	BSE, CBE and RS	Responsibilities at home Lack of money Being married Lack of access to health information	Fair

*A* Individual (personal) factor, *B* Provider (health-system) factor, *BSE* Breast self-examination, *CBE* Clinical breast examination, *RS* radiological screening (mammography and ultrasonography), *CW* community women, *HCWs* Healthcare workers, *CDs* communicable diseases, *NCDs* non-communicable diseases

**Table 3:** Characteristics of included quantitative studies

First author	Year	Country	Study design	Study focus	Sample size (n)	Study population	Data collection method	BCS method	Factors identified			Overall quality
Abay	2018	Ethiopia	Cross-sectional	Assess BSE practice and associated factors	400	Women aged 20–70 years	Interviewer-administered questionnaire	BSE	Perceived confidence to do BSE [AOR = 5.32; 95% CI (1.89–14.95)] Perceived susceptibility to develop BC [AOR = 3.79; 95% CI (1.74–9.74)]			Fair
Abeje	2019	Ethiopia	Cross-sectional	Identify factors associated with the awareness and practices of BCS	633	Women attending maternal and child health care services	Questionnaire	BSE, CBE, RS	Attended tertiary School [AOR = 4.00; 95% CI (1.48–10.86)] Lack awareness of BC [AOR = 0.01; 95%CI (0.00–0.03)]			Fair
Ameer	2014	Ethiopia	Cross-sectional	Assess the knowledge, attitude and practice of BSE	126	Female medical students at Haramaya University	Self-administered questionnaire	BSE	No signs or symptoms Forgetfulness Fear of detecting abnormality Lack of privacy			Fair
Antabe	2020	Kenya	Survey	Know determinants of utilization of BCS	14,734	Women who answered questions regarding BCS in the 2014 Kenya Demographic and Health Survey	Face-to-face interview	BSE, CBE, RS	Lower levels of education Single women Unemployed Rural		Poorer women Uninsured Aged 15–19	Good
Assefa	2021	Ethiopia	Cross-sectional	Measure BCS practice and associated factors	610	Community-women aged 20–70 years in urban settings of SNNPR, Ethiopia		BSE, CBE, RS	Positive attitude towards BCS (AOR = 3.0; 95%CI: 1.63–5.56) Educational status of college and above (AOR = 3.8; 95%CI: 1.25–11.48) Family history of BC (AOR = 3.7; 95% CI: 1.73–7.96) Awareness about BCS methods (AOR = 3.0; 95%CI: 1.46–6.22) Knowing someone screened for BC (AOR = 2.2; 95%CI: 1.10–4.38) Recommendation by health professionals for BCS (AOR = 5.0; 95%CI: 2.35–10.68)			Good
Atuhairwe	2018	Uganda	Survey	Effect of BC knowledge on the adoption of BC prevention modalities	414	Women in Kyadondo County	Questionnaire	BCS	Educational level Knowledge of BC and BCS			Fair
Ayugi	2020	Uganda	Cross-sectional	Determine factors associated with BC awareness, BSE, CBE, and other modalities for screening and early BC detection	98	Businesswomen ≥ 18 years in Gulu’s main market	Face-to-face questionnaire interviews	BSE, CBE and mammography	BSE –Vendor (OR = 1.989) –Work duration (1–10 years) (OR = 3.149) –University graduates (OR = 2.638)	CBE –Vendor (OR = 2.747) –Work duration (1–10 years) (OR = 2.347) –University graduates (OR = 2.347) No factors are associated with the use of mammography		Good
Azage	2013	Ethiopia	Cross-sectional	Determine BSE and identify factors associated with BSE	395	HEWs in West Gojjam Zone	Self-administered questionnaire	BSE	Discussion with families on BSE importance [AOR: 5.51, 95% CI: (3.45, 8.79)] Practice CBE [AOR: 2.69, 95%CI: (1.31, 5.52)]			Fair
Birhane	2017	Ethiopia	Cross-sectional	Assess the magnitude of practices of BSE and associated factors	420	Undergraduate female students at Debre Berhan University	Self-administered questionnaire	BSE	Knowing how to perform BSE [AOR 11.2, 95% CI (4.542–27.607)] Knowing when to perform BSE [AOR 3.5, 95% CI (1.620–7.593)] Knowing positions to perform BSE [AOR = 2.3, 95% CI (1.104–4.599)] Perceived benefit of BSE [AOR = 6.8, 95% CI (1.640–28.509)]			Fair
Birhane N	2015	Ethiopia	Cross-sectional	Assess predictors of BSE	315	Female teachers in Kafa Zone	Self-administered questionnaire	BSE	Perceived susceptibility, [AOR = 1.95 95%CI (1.44–2.63)] Perceived severity [AOR = 1.24, 95%, CI (1.11–1.46)] Perceived net benefits [AOR = 1.1, 95%CI, (1.03–1.20)]			Fair
Bushakhala	2016	Kenya	Cross-sectional survey	Ascertain what distinguished women who chose to participate in CBE screening from those who did not participate in our setting	1,061	Women in Uasin Gishu, Nandi, and Mount Elgon who were invited to participate in CBE	Questionnaire + interview	BSE and CBE	CBE –Being older –Employed, –BC knowledge –Do BSE I–Not worried about the doctor’s time –a longer time ta o health facility	BSE –being younger –Employed –Better education –Family history of BC		Fair
Dagne	2019	Ethiopia	Cross-sectional	Determine BSE practice and identify associated factors	421	Female workers in Debre Tabor Town public health facilities	Face-to-face interview questionnaires	BSE	Knowledgeable female workers (AOR 5.74, 95% CI: 2.3–14.4) Family history of BC (AOR 6.5, 95% CI: 1.54–21.4) Self-efficacy in practising BSE (AOR 4.7, 95% CI: 1.84–12.11)			Fair
Dagne I	2019	Ethiopia	Cross-sectional		370	Female healthcare professionals at Dire Dawa	Self-administered structured questionnaire	BSE	Education 1^st^degree and above [AOR = 3.14, 95% CI (1.98, 6.21)] Clinical experience ≥ 10 years [AOR = 2.6, 95% CI (1.71, 6.23)] Positive attitude [AOR = 1.9, 95% CI (1.24, 8.04)] Good knowledge [AOR = 4.5, 95% CI (2.33, 12.34)]			Fair
Delie	2012	Ethiopia	Cross-sectional	Assess the knowledge about BC risk factors, breast screening methods and practice of breast screening	440	Female Healthcare Professionals Working in Governmental Hospitals	Questionnaire	BSE, CBE, and mammography	BSE; Marital status CBE; Age, marital status, educational level, type of profession and work experience RS; Age, marital status, educational level, work experience and type of profession			Fair
'Desta	2018	Ethiopia	Cross-sectional	Assessing the knowledge, practice and factors contributing to BSE	200	Female College Students	Interviewer administered questionnaires	BSE	Urban residents (AOR = 4.19; 95%CI = 1.63, 10.77; *P* = 0.003) Clinical year students (AOR = 7.24, 95% CI = 3.85, 13.59, *P* < 0.001)			Good
Dibisa	2019	Ethiopia	Cross-sectional study	Assess BCS practice and its associated factors	422	Women in Kersa district	Self-administered questionnaire	BSE	Age ≥ 26 years (AOR = 2.3;95% CI:1.4,3.7) Knowledge on BCS (AOR = 2.8; 95% CI: 1.2, 6.5) Good knowledge about BC (AOR = 3.4;95% CI: 1.3, 9.4)			Fair
Dinegde	2020	Ethiopia	Cross-sectional	Assess the knowledge and practice of BSE	381	Female undergraduate students at Addis Ababa University	Self-administered questionnaire	BSE	Age Knowledge of BSE			Fair
Elsie	2010	Uganda	Descriptive cross-sectional	Assess the knowledge, attitudes and practices of women about cancer and mammography as well as identify potential barriers	100	Women reporting to the Radiology department	Interviewer-administered questionnaires	Mammography	More than primary education (OR 3.79, 95% CI 1.51–9.43) Being employed (OR 6.9, 95% CI 1.46–333.21)			Poor
Getu	2016	Ethiopia	Cross-sectional	Assess BSE practice	407	Female undergraduate students in Addis Ababa University	Self-administered questionnaire	BSE	Family history of BC (AOR = 2.332; 95% CI, 1.009–5.389, *P* = 0.048) Good attitude (AOR = 4.68; 95% CI, 2.411–9.067, *P* ≤ 0.001) Good knowledge (AOR = 12.422; 95% CI, 5.478–28.167, *P* ≤ 0.001)			Fair
Gochole	2020	Ethiopia	Cross-sectional	Assess the BSE practice and associated factors	150	First-year Health Science Female Students of Ambo University	Self- administered questionnaire	BSE	Family history of BC (AOR = 2.12; 95% CI, 1.09–3.95, *P* = 0.044) Good knowledge about BSE (AOR = 9.5; 95% CI, 5.5–18.8, *P* = 0.002)			Poor
Hailu	2016	Ethiopia	Cross-sectional	Assess the level of knowledge and attitude towards BC and practice of self-breast examination	760	Female students, in Mekelle University	self-administered questionnaire	BSE	Not having breast complaints Not knowing how to do BSE			Fair
Jembere	2019	Ethiopia	Cross-sectional	Assess practice of breast self-examination and associated factors	180	Female nurses of Hawassa University Comprehensive Specialized Hospital	Self- administered questionnaire	BSE	Higher Education (AOR = 2.91 95% CI (1.74, 4.85)) Family history of BC (AOR = 5.2 95% CI (2.34,8.15))			Fair
Joyce	2020	Uganda	Cross-sectional	Assess the knowledge and practice of BSE	386	Female clients ≥ 18 years at the Family planning unit, Early Infant Diagnosis clinic and Out-patient department of Mbale Regional Referral Hospital	Interviewer administered questionnaire	BSE	Urban address Level of education Occupation Religion Knowledge of BSE			Fair
Kifle	2016	Eritrea	Cross-sectional	Understanding the knowledge level and practice of BSE	380	Female college students in seven colleges in Eritrea	Self-administered Questionnaire	BSE	Lack of knowledge			Poor
Kassa	2017	Ethiopia	Cross-sectional	Assess the knowledge of BC and BSE	423	Female Students in Rift Valley University, Adama campus	Self-administered Questionnaire	BSE	Enrolment in health sciences [AOR 3.696 (2.199,6.23)]			Fair
Legesse	2014	Ethiopia	Cross-sectional		845	Women household heads in the town	Semi-structured questionnaire	BSE	College/university education (AOR = 4.65, 95% CI = 2.19–9.86) History of breast problems (AOR = 2.28, 95% CI; 1.14–4.33) BC knowledge (AOR = 3.02, 95% CI = 2.20—10.67)			Fair
Lera	2020	Ethiopia	Cross-sectional	Assessed BSE and associated factors	629	Women in Wolaita Sodo, 20-65 years	Interviewer-administered questionnaire	BSE	Previously heard of BSE (AOR = 6.36, 95% CI: 3.72, 10.71) Employed (AOR = 3.13, 95% CI: 1.14, 8.58) University students (AOR = 3.73, 95% CI: 1.19, 11.73)			Fair
Mekuria	2020	Ethiopia	Cross-sectional	Assess the level of practice of BSE and its associated factors	239	Secondary School Female Teachers in Gammo Gofa Zone	Self-administered questionnaire	BSE	Ever heard about BSE [AOR = 2.26, 95% CI (1.07, 4.77)] Knowledge on BSE practice [AOR = 2.84, 95% CI (1.41, 5.72)] Less perceived barrier to BSE [AOR = 2.62, 95% C. I (1.26, 5.46)] High perceived confidence to practice BSE [AOR = 3.63, 95% C. I (1.79, 7.39)] Having motivation to practice BSE [AOR = 3.29, 95% CI (1.15, 9.45)] Married [AOR = 4.098, 95% CI (1.644, 10.219)]			Good
Mereta	2019	Ethiopia	Cross-sectional	Assess the level of BSE and its associated factors	634	Community-women aged 20-64 years at Arbaminch Zuria district	Interviewer-administered questionnaire	BSE	Practice BSE – fear of developing cancer and early detection, breast problem Not practising BSE – not knowing how to perform BSE, not knowing its importance, not having breast symptoms			Fair
Mihret	2021	Ethiopia	Cross-sectional	Assess knowledge and practice on BSE and its associated factors	384	Undergraduate Female Students in the University of Gondar	Interviewer-administered structured questionnaire	BSE	Family history of BC (AOR = 7*:*14; 95% CI: 1.75, 25) Discussion with someone on BSE (AOR = 3*:*85; 95% CI: 1.82, 8.33) Good knowledge on BSE (AOR = 12*:*02; 95% CI: 5.97, 24.20)			Good
Minasie	2017	Ethiopia	Cross-sectional	Assess BSE and identify factors associated with BSE	281	Health extension workers in Wolayta zone	Interviewer administrated questionnaire	BSE	Younger women 25–29 years and 30–34 years age 0.065(0.017–0.248), 0.070(0.015–0.328)] Perceived benefit (AOR = 3; 95%CI of [1.5–6.5]) High perception for BC [AOR = 5.0(2.0–10.5)] Support from spouse/partner (AOR = 3; 95% CI of [1.5–8.75])			Fair
Morse	2014	Tanzania	Survey	Assess BC knowledge, beliefs, and practices	225	Women presenting to district hospitals for outpatient medical care	verbal survey	BSE, CBE, RS	Facilitators—knowledge of the procedure, available instruction sheet, and the opportunity to attend an awareness program Barriers – lack of knowledge about the procedure (46.2%), forgetfulness (30.0%), and feeling overworked/too busy (30%)			Fair
Natae	2015	Ethiopia	Cross-sectional	Assess awareness, attitude and practice of BSE for BCS	305	Undergraduate female students of Ambo University	Self-administered questionnaires	BSE	Lack of knowledge of how to perform Having no breast problem			Poor
Negeri	2017	Ethiopia	Cross-sectional study	Assess the magnitude of BSE practise and its associated factors	300	Female health professionals working in public health facilities	Self-administered questionnaire	BSE	Personal history of BC (AOR = 4.7, 95%CI: 1.32, 17.07) Knowledgeable (AOR = 4.2, 95%CI: 1.36, 5.65) Teaching BSE (AOR = 5.2, 95%CI: 2.33, 8.14) Positive attitude (AOR = 3.8, 95%CI: 2.11, 9.17)			Fair
Ngi’nda	2019	Tanzania	Descriptive cross-sectional	Describe the knowledge and practices on BC and associated challenges	130	Community-women ≥ 35 years in Morogoro Rural District	Interviewer administered questionnaire	BSE	Lack of knowledge Regarding BSE as not important Regarding themselves as not at risk			Fair
Oguta	2022	Kenya	Cross-sectional	Assess the knowledge and practice of BSE for the early detection of BC	398	Health facility nurses and women attending an antenatal and maternal clinic at JOOTRH, Kisumu	Semi structured questionnaires and Key informant interviews	BSE	College education (AOR = 3.25, 95%CI [1.03–10.25], *p* = 0.044) Participants who were not married/cohabiting (AOR = 0.60, 95%CI [0.36–0.99], *p* = 0.048)			Poor
Scheel	2017	Uganda	Survey	Provide information about downstaging practices and breast health messaging preferences	401	Rural and urban women from Kampala, Rakai district and Kooki county, ≥ 25 years	Questionnaire	BSE and CBE	Urban participants (BSE and CBE) More than a primary school education (BSE) Regular care at the health centre (CBE) Previous BC education (BSE and CBE)			Fair
Shallo	2019	Ethiopia	Cross-sectional	Assess the magnitude of BSE practice and associated factors	340	Female healthcare Workers in West Shoa Zone Oromia regional state	Self- administered questionnaire	BSE	Level of education Attitude towards BSE Knowledge of BSE			Fair
Sharp	2019	Uganda	Survey	Prevalence of patient-related barriers and their associations with BC detection practices	401	Urban women from Kampala and rural women from Kakuuto and Kooki counties	Questionnaire	BSE, CBE and breast ultrasound	Rural residency Poor social support Fear Economic barriers to accessing care, Knowledge deficits			Good
Taklual	2021	Ethiopia	Cross-sectional	Assess the practice of BSE	342	Female students at Debre Tabor University	Self-administered Questionnaire	BSE	Information about BSE ((AOR) = 7.21, 95% CI: (2.46, 21.15)) Perceived susceptibility (AOR = 14.18, 95% CI: (4.00, 50.48)) Self-efficacy (AOR = 3.07, 95% CI: (1.09, 8.70)) Net benefit of BSE (AOR = 7.75, (1.56, 38.55))			Fair
Tewabe	2016	Ethiopia	Cross-sectional	Assess the knowledge and practice of BSE	459	Female undergraduate students in Bahir Dar University, Ethiopia	self-administered semi-structured questionnaire	BSE	Negligence Forgetfulness Lack of knowledge			Poor
Terfa	2020	Ethiopia	Cross-sectional	Assess the level of BSE knowledge and practice	724	Women of childbearing age group in Jimma town	Interviewer administered questionnaire	BSE	Women aged ≤ 35 years (AOR = 2.072/1.146, 3.747/0.016) Employed women (AOR = 3.936/1.497, 10.351/0.005) Family history of BC (AOR = 4.167/2.358, 7.364/0.000) Women earning ≥ 40 USD (AOR = 5.570/1.557, 19.922/0.008)			Good
Urga	2021	Ethiopia	Cross-sectional	Assess BSE practice and associated factors	420	Women attending family planning service in Modjo public health facilities		BSE	Tertiary level education [AOR: 2.14; 95% CI: (1.45, 6.74)] Knowledge about BSE [AOR: 4.32; 95% CI: (1.81, 10.81)] Positive attitude towards BSE [AOR: 2.7; 95% CI: (1.03, 6.91)]			Good
Wachira	2014	Kenya	Descriptive survey	Explore perceptions of barriers to participation in BCS	733	Women not attending BCS services at three AMPATH sites	Interviewer + self-administered questionnaires	BSE, CBE, mammography	–No barriers to BSE CBE– an embarrassment of CBE, fear of screening outcome, busy schedules, low personal risk, negative social influence Mammography–high cost of medical care, perceived poor quality of health services, long distance to the health facility, long queues in health facilities			Fair
Wurjine	2019	Ethiopia	Cross-sectional	Assess knowledge, attitude and practice of BC and BC early detection methods	389	Female health professionals	Self-administered questionnaire	BSE, CBE, mammography	BSE–marital status and profession CBE–aged 29–38 vs > 49 [AOR = 0.36; 95%CI (0.002–0.54)] –Degree vs diploma holders, [AOR = 1.80; 95%CI (1.06–3.07)] –Married vs single, [AOR = 3.39; 95%CI (1.97–5.80)] –Family history of breast problem [AOR = 0.19; 95%CI (0.05–0.72)]			Good
Zeru	2018	Ethiopia	Cross-sectional	Assess knowledge, attitude and practices about BSE and associated factors	453	Urban Health Extension Workers in Addis Ababa	Self-administered questionnaire	BSE	Work experience ≥ 1 year (AOR: 2.8; 95%CI: 1.1, 5.7) Familiarity with people with a history of BC (AOR: 1.7; 95%CI: 1.1, 2.6) Perceived susceptibility (AOR: 1.8; 95%CI: 1.2, 3.0) Knowledge of BSE (AOR: 2.3; 95%CI: 1.4, 3.7)			Fair

*A* Individual (personal) factor, *B* Provider (health-system) factor, *SNNPR* Southern Nations Nationalities and People’s Region, *JOOTRH* Jaramogi Oginga Odinga Teaching and Referral Hospital, *BSE* Breast self-examination, *CBE* Clinical breast examination, *RS* radiological screening (mammography and ultrasonography), *HEWs* Health extension workers, *HCWs* Healthcare workers

### Qualitative studies

Four of the studies used focus group discussions, and one used in-depth interviews. The sample size ranged from 24–80. They all assessed barriers/facilitators for all BCS methods, the study population being community women (with and without BC) and health care providers. The quality of the included studies ranged from medium (*n* = 3) to high (*n* = 2). Table 2 shows the study characteristics of the included qualitative studies.

### Quantitative studies

Of the 46 included studies, 39 were cross-sectional studies, and seven were surveys. Questionnaires were employed in the studies. The sample size ranged from 98 [24] to 14,734 [25]. Most studies did not indicate a participant’s age limit, though it ranged from as low as 15 years of age [22] to as high as 70 years of age [8, 26].

Studies that focused on breast self-examination (BSE) were 32. Three studies focused on both BSE and clinical breast examination (CBE), one study focused on mammography alone, while ten involved three methods of BCS (BSE, CBE, and mammography). Study population included female university students (*n* = 13), community women (*n* = 23), and healthcare workers (*n* = 11). Quality of included studies ranged from poor (*n* = 6), fair (*n* = 29) and good (*n* = 11). Table 3 shows the study characteristics of the included quantitative studies.

### Synthesis results

**Fig. 2:**
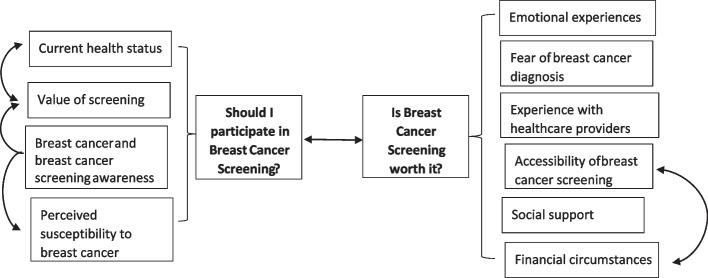
Interaction of themes and subthemes

Based on the response of the participants with regards to barriers and/or facilitators of BCS uptake, two major themes were identified; (a) should I participate in screening? and, (b) is breast cancer screening worth it? (Table 4 shows themes and subthemes). Figure 2 shows the interaction of these themes and the subthemes influencing them.

**Table 4:** Thematic themes and subthemes

Theme	Subtheme	Qualitative studies supporting the subtheme	Quantitative studies supporting the subtheme
Should I participate in BCS?	Current health status	[27, 28]	[12, 26, 29–41]
	Value of screening	[27, 42, 43]	[26, 30, 32, 34, 39, 44–48]
	BC and BCS awareness	[27, 28, 42, 43, 49]	[12, 13, 15, 16, 26, 31, 32, 36, 40, 44, 46, 48, 50–60]
	Perceived susceptibility to BC	[42, 42]	[11, 13, 26, 33, 36, 39, 41, 44, 51, 55, 57, 60, 61]
Is BCS worth it	Emotional experiences	[27, 28, 43, 49]	[62]
	Fear of BC diagnosis	[27, 43, 49]	[35, 54, 58, 62]
	Experience with healthcare providers	[28, 43, 49]	[62, 63]
	Accessibility of BCS services	[28, 42, 43, 49]	[62–64]
	Social support	[27, 43, 49]	[13, 15, 25, 26, 32, 33, 39, 41, 45, 51, 58, 60, 61, 65, 66]
	Individual and family financial circumstances	[27, 28, 43]	[14, 15, 24, 25, 33, 41, 53, 55, 60, 64, 65]

### Theme 1: Should I participate in the screening?

The first major theme was related to the relevance and importance of BCS as seen by the women, and ultimately whether they should participate in it. Women’s participation in BCS was largely influenced by four things (sub-themes) – current health status, perceived risk of having BC, awareness of BC and BCS, and perceived benefit in screening.

#### Current health status

Women explicitly discussed the absence of breast symptoms and being generally healthy as an indication that screening was not required [27, 28, 35, 37, 40, 54, 57, 59]. Additionally, in the study by Muthoni and Miller (2010), some women also responded that they would be wasting the provider’s time if they asked for screening without any complaint [27].

#### Awareness of BC and BCS

A vast majority of the women confessed to having no idea about the different methods of BCS, how to perform BSE, how often to have a breast examined and where to be examined [9, 26, 28, 30, 42, 43, 49, 56]. This lack of information seemed to affect rural women more as they lack sources of information about BC and BCS [28, 42, 43]. Multivariate analysis showed those with poor knowledge and awareness of BC and BCS were less likely to undergo BCS [26, 36, 48, 51].

Further, those with higher levels of education were more likely to be knowledgeable about BC and BCS and also more likely to undertake BCS compared to those with lower levels of education [14, 15, 24, 38, 50, 61, 64, 67]. This could be related to education providing an avenue for learning health issues like BCS [45]. Additionally, older women were shown to be more likely to participate in BCS compared to younger women, as they were more knowledgeable on health issues and also aware of the relationship between age and BC susceptibility [16, 25, 45, 64].

#### Perceived risk of having breast cancer

There was generally a low self-susceptibility to BC due to various socio-cultural beliefs on the causes of BC [11, 42, 44, 45, 47, 60, 62]. These beliefs included BC being a result of sin, promiscuity, curses, and deviation from the socio-cultural norm [42, 43]. A 39 year-old participant from a study by Agide et al., (2019) [42] said,

*“As to me, doing good or bad acts will determine the occurrence of a disease. A good act leads to good health and a bad leads to disease…”* [42].

Family history was often indicated as a risk factor for BC, and some women interpreted its absence as an indication of being at lower risk of having BC and thus no value in BCS [27, 39, 41, 51, 55, 61].

#### Value of screening

Some women expressed the value of BCS as it could identify problems early and allow appropriate treatment, it would increase their chances of survival and decrease treatment costs if caught early, and it would also help prepare their families for the possibility of death and thus make it easier to cope emotionally with the condition [27, 43]. Women who perceived BCS to be beneficial had odds higher odds of practice [32, 34, 39, 44].

Another group of women saw no advantage in undergoing BCS. Most women viewed BC as a terminal disease with no advantage in early detection practice [27, 42, 43]. Others believed that cancer is a result of supernatural causes and there is therefore no point in screening [42, 43, 57]. This view prevented some from undergoing screening as they relied on prayers as a means to prevent diseases and some religious organizations prohibited their followers from going to the hospital [43]. A 22-year-old asymptomatic participant from Agide et al., (2019) [42] stated,

“*I think it is common for all of us to perceive BC as it is not a curable disease. Since the cancer treatment is found outside the country, it is not affordable and the only option is death. For the question, you asked as ‘do women prefer to use screening as a primary prevention method?’ In my opinion, I don’t think so*” [42].

### Theme 2: Is breast cancer screening worth it?

The second theme is related to women’s burden – both physically and emotionally in undergoing breast cancer screening. This relates to experiences in undertaking BCS.

#### Emotional experiences

Having a diagnosis of BC is a very crippling emotional experience as described by several women [27, 28, 42, 49]. They expressed emotions of low self-esteem and anxiety if they were found to have BC from screening, and also doubts with regards to the quality of life after diagnosis and treatment and the state of their families and children if they become debilitated or die [28, 43, 46]. Wachira et al., also noted other women are embarrassed to undergo CBE [62].

### Fear of BC diagnosis

Another emotional experience acting as a barrier to women opting to undertake BCS was fear of diagnosis [35, 54, 58, 62]. This fear stemmed from the possibility of having to undergo surgery and the possibility of dying if BC was diagnosed [43, 49]. A key informant in a study by Ilaboya et al., (2018) [28] stated,

*“So many people even fear to go for screening because they say ‘why go? Because if they discover cancer I am doomed to die.’ So they have that feeling that once detected it won’t be cured” *[28]*.*

Fear was also related to the social threat of being divorced, and being infertile if they underwent mastectomy due to the disease [43, 49]. Such emotional experiences were described as barriers to screening even among women with a positive attitude toward BCS [27, 43].

#### Experience with healthcare professionals

Women’s motivation for screening was often shaped by the quality of their interaction with the healthcare workers. Some women recalled negative encounters, with poor communication cited as the reason they did not pursue further screening [27, 43]. Women preferred to receive care from tertiary institutions rather than primary care facilities, as they felt they received better care in tertiary facilities [28, 63]. Ilaboya et al., (2018) [28], also reported a general perception among community participants that the healthcare workers in primary care settings were inexperienced [28]. This was shown by focus group discussions among healthcare workers that revealed a low level of awareness about BC and BCS [28, 43].

#### Accessibility of BCS services

Several women reported they were unable to access BCS services due to long distance to health facilities [15, 25, 49, 64, 67], absence of screening services in primary care settings [28, 49] and also the high cost of services [27, 42, 58, 62]. For others, screening was seen as inaccessible due to long waiting times in health facilities that deterred them from pursuing BCS [28, 43, 62].

#### Social support

Some women described how screening was another demand on their time and often competed with other daily tasks [27, 56, 62]. Various women discussed how their duties in the family prevented them from health-seeking activities such as educational forums and screening programs [27, 43, 49]. Married women and single mothers voiced their concerns about how overwhelming their duties can be [27, 43, 49].

Married women in particular cited their husbands as prohibitors to health activities due to the husband’s position as the “*head of the home*” [27, 43]. Despite this, various multivariate analyses showed married women to have better odds of practicing BCS compared to those not married [25, 32, 41, 58, 65]. Of note, Minasie et al., (2017) and Sharp et al., (2019), reported that married with poor social support have lower BCS practices [45, 58].

### Individual and family financial circumstances

Finances affected screening services in a variety of ways including preference of screening method, access to screening services (service cost and transport cost) and perceived benefit of screening (economic advantage of screening) [27, 28, 42, 43, 49].

Women were less likely to undertake CBE and mammography due to their cost and preferred BSE as expressed by a participant’s preference for BSE because,

“*…it is convenient as it doesn’t cost anything*” [11].

Expenses to access screening services (CBE and mammography) were rooted in transport-related costs, and the cost of the services [12, 25, 27, 43, 49, 58]. Others saw no direct financial advantage in undergoing screening [28, 43, 49] as is illustrated by a 60 year old rural participant,

*“I am not going for BCS. They are not going to give me food so let me go to my garden and dig. Am I going to eat from there? Do they eat cancer? I don’t want to do it”* [28].

Perceived financial benefit of BCS could be the reason why self-employed and unemployed women were less likely compared to those who were employed to undergo BCS [11, 43, 47, 53, 55]. It could also be attributed to income level, as those with lower income are less likely to undergo BCS compared to those with better income [24, 33, 34, 60, 65]. Additionally, women with a longer duration of employment and those with employed husbands were more likely to participate in BCS [12, 13].

## Discussion

This review examined the evidence for factors influencing BCS practice among women in Eastern Africa (EA). It has generated an understanding of how BCS is experienced by women in EA and reveals findings that are important for expanding BCS in the region and other similar countries around the world. The main finding was that lack of knowledge and awareness about BC and BCS were the key barriers to BCS irrespective of country, study population or methodology. Two other important observations made in this study were the effect of social roles among women in EA and the accessibility of BCS services.

An appreciation of how Eastern African women perceive their social roles helps understand how their roles affect screening practices. This is because several women reported a lack of support in their household duties as a barrier to attending health forums and screening activities [27, 28, 43]. Women’s role in this region as mothers and wives is to act as a caretaker of the family, which means they sometimes put their family’s needs above their own [2, 68]. Studies in East Africa have shown women to have lower autonomy on matters of their health [69–71]. Lack of home support and autonomy in health decisions has also been reported in studies done in Asia and among Asians, Hispanics and Blacks living in high income countries [72–76]. Women globally need to be supported and encouraged to participate in such screening activities that can have a profound impact on their livelihoods.

Another observation made is the need for resource allocation and facilitation of educational programs at the patient and provider levels. The effect of lack of knowledge identified included women who do not know screening is required, do not know where to go for screening, do not know how to perform BSE, have limited knowledge about screening methods, and lack knowledge about BC (cause, signs and symptoms, treatment and prognosis). Poor knowledge regarding breast cancer has also been observed in other countries in Africa, Asia, Europe and USA, and this has consistently been indicated as a barrier to participating in breast cancer screening [1, 15, 73, 74, 76–79]. In settings with limited resources like EA countries, the approach might focus on enhanced awareness and capacity building for breast evaluation [16, 80].

Studies done in other India and Mexico indicate low levels of cancer awareness even among those with higher education or socioeconomic status [81, 82]. However, the Global Breast Cancer Initiative indicates that with financial and educational investment to improve cancer literacy in LMICs, the public may be more likely to utilize screening programs [83]. Studies done in Africa and Asia have also shown increased utilization of breast cancer screening services among patients with higher levels of education and socioeconomic status [1, 15, 73–75]. Results from this suggest that providers, especially those from primary care settings require more rigorous training programs for early detection methods and guidelines, this also includes training on patient-provider interactions [17]. Pace and Shulman (2016) suggested quality control and ongoing training of practitioners in CBE must be an essential part of a CBE early detection program [16].

Another major component is that screening services are less accessible, especially in rural settings. Screening services are expensive and unavailable in most African countries, this is contrast to studies done in Asia, Europe and America where breast cancer screening services are more available though not readily accessible due to cost [1, 15, 72–79]. Decentralization of screening services tailored to EA rural areas will enhance the availability of screening services [84]. Pace and Shulman (2016) reported that even without systematic screening or early detection campaigns, the development of more accessible health facilities leads to a shift in the stage distribution of breast cancer over time [16]. These can include the use of mobile clinics in areas with limited healthcare infrastructure, and subsidized or free screening services [17].

Based on this review, several priorities need to be considered for the development and implementation of breast cancer screening in EA. These include financial and resource allocation to;

1. Community education programs to facilitate screening uptake
2. Enhanced training for healthcare providers particularly those in the primary care settings
3. Decentralization of screening activities to meet the needs of under-resourced populations, especially in rural areas.

The main challenge in screening interventions in Sub-Saharan Africa is the gap between conducting a good screening program and appropriate follow-up with diagnosis and treatment [16, 17]. Strategic investments in cancer control and implementation to ensure universal access to cancer are required to achieve the Sustainable Developmental Goals [85]. The World Health Organization highlighted financing, partnership, legislative frameworks, policy integration, leadership and advocacy, and development and allocation of human resources as key aspects to facilitate effective policy development [86].

### Strengths of the review

To our knowledge, this is the first review that systematically summarized studies on factors influencing BCS among women in Eastern Africa. We performed an extensive systematic search of the literature with no limitation on time. We included both qualitative and quantitative studies investigating BCS uptake and associated factors among EA countries. A thematic synthesis of the factors influencing breast cancer screening uptake was done together with a multisource synthesis of qualitative and quantitative data. Quality appraisal of the included studies was done, and no study was refuted based on quality.

### Limitations of the review

The findings from this review are subject to the following limitations. First, we found no data from Burundi, Comoros, Djibouti, Rwanda, Seychelles, Somalia, South Sudan and Sudan, we, therefore, have no insight into these countries. Secondly, there was a variation in methodology among quantitative studies which precluded meta-analysis of factors associated with screening practices. Meta-analysis would predict the effect size of each factor. Third, since the literature search and selection process was done in English, relevant articles in other languages were not identified. Also, exclusion of unpublished reports, review articles, conference abstracts and thesis may have omitted relevant information. Lastly, we did not assess for publication bias.

## Conclusion

In this review, many factors were identical irrespective of the country where the study was done. Improving knowledge and awareness among both the public and providers may be the most effective strategy to improve BCS in Eastern Africa. Breast health awareness should be promoted, effective training of relevant staff in CBE should be done, opportunistic CBE screening has to be encouraged and the feasibility of mammography has to be evaluated. There is a need to strengthen political will toward these core policy features to develop robust national breast cancer screening programs. Increased financial, human, and research efforts are also needed to sufficiently address the existing and increasing need for cancer services.

Overall, this review has highlighted that whilst there is a range of publications reporting the practice of BCS and associated factors in women in EA, there remains a significant scant body of evidence describing BCS practices in this region as most identified studies came from Ethiopia, and also majority focused on BSE. This review can be used as a starting point for further research into this problematic area of primary public health practice.

### Supplementary Information

**Additional file 1.** Search Strategy for MEDLINE

**Additional file 2.** Quality assessment tool for the included studies

**Additional file 3.** 

## Data Availability

The datasets used and/or analysed during the current study are available from the corresponding author on reasonable request.

## Abbreviations

BC: Breast cancer
BCS: Breast cancer screening
BSE: Self breast examination
CBE: Clinical breast examination
EA: East Africa
HICs: High income countries
LMICs: Low- and middle-income countries
RS: Radiological screening
SSA: Sub-Saharan Africa
