# Supplementary material for: Barriers and enablers of breast cancer screening among women in East Africa: a systematic review
Source: BMC Public Health. 2023 Oct 4;23:1915. doi: 10.1186/s12889-023-16831-0 (PMC10548570; doi:10.1186/s12889-023-16831-0)
Supplement: Supplementary file 1 — Additional file 1. Search Strategy for MEDLINE [file 12889_2023_16831_MOESM1_ESM.docx]

## Additional file 1

## Search Strategy for MEDLINE

| **A** | **B** | **C** | **D** |
| --- | --- | --- | --- |
| (Breast or Mammary) SH  AND  Neoplasms (SH)  neoplasm* (free text)  cancer* (free text)  tumor* (free text)  malignancy * (free text) | Africa, Eastern* (Burundi, Comoros, Djibouti, Eritrea, Ethiopia, Kenya, Rwanda, Seychelles, Somalia, South Sudan, Sudan, Tanzania, Uganda) | Perception (SH)  Social Perception (SH  perception* (free text)  social perception* (free text)  opinion* (free text)  Attitude to Health (SH)  attitude* (free text)  social value* (free text)  social norm* (free text)  Culture (SH)  belief* (free text)  understanding* (free text)  language* (free text)  communicat* (free text)  fear* (free text)  mistrust (free text)  trust (free text)  cultur* (free text)  relig* (free text)  knowledge* (free text)  barrier* (free text)  embarrass* (free text)  fatalism (free text)  fatalistic (free text)  income (free text)  socioeconomic* (free text)  depriv* (free text)  educat* (free text)  poor* (free text)  poverty (free text) | Screening (SH)  Early Diagnosis (SH)  “Early detection of cancer” |

**Search done was A and B and (C or D)**
