# Supplementary material for: Barriers and enablers of breast cancer screening among women in East Africa: a systematic review
Source: BMC Public Health. 2023 Oct 4;23:1915. doi: 10.1186/s12889-023-16831-0 (PMC10548570; doi:10.1186/s12889-023-16831-0)
Supplement: Supplementary file 2 — Additional file 2. Quality assessment tool for the included studies [file 12889_2023_16831_MOESM2_ESM.docx]

# Additional file 2.

# Quality assessment tool for the included studies

| Screening questions for all study types | Qualitative studies | Quantitative studies |
| --- | --- | --- |
| -Clear objectives or questions?  -Does the collected data address the research question?  -Appropriate design?  -Justification of setting?  -Sampling method explained?  -Appropriate selection process?  -Adequate discussion?  -Research implications?  -Sources of bias?  -Ethical approval? | -Are the sources of collected data relevant to address the research question?  -Is the process of data analysis relevant to address the research question?  -Was appropriate consideration given to how findings relate to the context of the study?  -sources of bias?  -Clear themes for synthesis?  -Sufficient data to support findings?  -Consideration of contradictory findings?  -Credibility of findings? | -Is the sampling strategy relevant to address the research question?  -Is the sample representative of the population under study?  -Explicit data collection?  -Description of analysis process  -Appropriate statistical analysis?  -Sufficient data analysis?  -Identification of conditions?  -Measurement of conditions?  -Is there an acceptable response rate?  -Appropriate consideration is given to sources of bias? |

Each criterion was given a score from 0-2 based on the author’s subjective judgement. These were then summed and an assessment of the overall quality of a particular study was ranked as “good”, “fair”, and “poor”. The quality score for quantitative studies ranged from 0-40 (0-20=poor, 21-30=fair, 21-40=high). The quality score for qualitative studies ranged from 0-36 (0-18=poor, 19-28=fair, 29-36=good). No studies were excluded as a result of the quality assessment, rather, the quality assessment contributed to the confidence of each finding.
