# Supplementary material for: Barriers and enablers of breast cancer screening among women in East Africa: a systematic review
Source: BMC Public Health. 2023 Oct 4;23:1915. doi: 10.1186/s12889-023-16831-0 (PMC10548570; doi:10.1186/s12889-023-16831-0)
Supplement: Supplementary file 3 — Additional file 3. [file 12889_2023_16831_MOESM3_ESM.docx]

## Additional file 3

## Narrative Synthesis of Quantitative Studies

**Overall Uptake of Breast Cancer Screening**

Overall screening practice ranged from 6.9-13.6% among community women in Ethiopia ([13](#_ENREF_13), [24](#_ENREF_24)) and 8-12% among community women in Kenya ([23](#_ENREF_23), [25](#_ENREF_25)). Included studies reported screening by mammography to have been conducted by 0-24.2% of the participants ([9](#_ENREF_9), [24](#_ENREF_24), [47](#_ENREF_47), [48](#_ENREF_48)). Clinical breast examination uptake ranged from 5.5-to 32.5% in Ethiopia ([9](#_ENREF_9), [13](#_ENREF_13), [24](#_ENREF_24), [27](#_ENREF_27), [47](#_ENREF_47), [48](#_ENREF_48), [51](#_ENREF_51)), 15-40% in Uganda ([11](#_ENREF_11), [22](#_ENREF_22), [56-58](#_ENREF_56)) and 6.2% in Tanzania (although regular participation was at 0.9%) ([55](#_ENREF_55)). Participants who reported to have ever done BSE ranged from 3.6% to 79.9% overall, though the regular practice of BSE ranged from 2.6%-37.9% ([8-13](#_ENREF_8), [26](#_ENREF_26), [28](#_ENREF_28), [30](#_ENREF_30), [33](#_ENREF_33), [34](#_ENREF_34), [36-39](#_ENREF_36), [41-52](#_ENREF_41), [54](#_ENREF_54)). In Ethiopia, regular BSE practice ranged from 2.6%-31%, although those who reported to have ever undergone BSE were 13.1%-79.9% ([8](#_ENREF_8), [10](#_ENREF_10), [13](#_ENREF_13), [26-52](#_ENREF_26)). Overall uptake of BSE was 27-66% in Uganda ([11](#_ENREF_11), [12](#_ENREF_12), [56-58](#_ENREF_56)), 40.7% in Kenya ([53](#_ENREF_53)) and 18.5-25.4% in Tanzania ([54](#_ENREF_54), [55](#_ENREF_55)).

Seventeen studies were done among community women. The included studies reported overall BSE practice to be 3.6% to 34.4% and regular practice to be 3.6-13.3% in Ethiopia ([8](#_ENREF_8), [9](#_ENREF_9), [13](#_ENREF_13), [24](#_ENREF_24), [33](#_ENREF_33), [35](#_ENREF_35), [39](#_ENREF_39), [51](#_ENREF_51)). In Uganda, overall BSE practice was 27-66% ([11](#_ENREF_11), [12](#_ENREF_12), [56-58](#_ENREF_56)), 18.5-25.4% in Tanzania ([54](#_ENREF_54), [55](#_ENREF_55)), and 40.7% in Kenya ([53](#_ENREF_53)). Also, we had eight studies done among university students that reported regular BSE practice to be 6.8%-25.3% and overall BSE practice was 15% to 44.7% ([28-32](#_ENREF_28), [36-38](#_ENREF_36), [41](#_ENREF_41), [43](#_ENREF_43), [45](#_ENREF_45), [50](#_ENREF_50)). Among healthcathe re workers, included studies reported regular practice of BSE to range from 14.4% to 49.5%, although overall practice of BSE was 35.5-79.9% ([10](#_ENREF_10), [27](#_ENREF_27), [40](#_ENREF_40), [42](#_ENREF_42), [46](#_ENREF_46), [48](#_ENREF_48), [49](#_ENREF_49), [52](#_ENREF_52), [63](#_ENREF_63)).

1. **Individual Related Factors**

**1a. Economic factors**

Twelve of the 46 included studies found an association between employment status and BCS practice. Women who were self-employed and unemployed were less likely compared to those who were employed to undergo BCS ([8](#_ENREF_8), [23](#_ENREF_23), [33](#_ENREF_33), [38](#_ENREF_38), [66](#_ENREF_66)). Also, women married to men who were employed had positive odds of undergoing BCS ([9](#_ENREF_9), [10](#_ENREF_10)). In addition to this, participants who were one year or more in their employment were more likely to practice BCS compared to those with lower duration of employment ([22](#_ENREF_22), [38](#_ENREF_38), [40](#_ENREF_40), [48](#_ENREF_48), [52](#_ENREF_52)).

Other economic factors that influenced BCS uptake were: income level (poorer women were less likely to have been screened for BC than richer women) ([9](#_ENREF_9), [10](#_ENREF_10), [22](#_ENREF_22), [23](#_ENREF_23), [57](#_ENREF_57)) and health insurance (those with no health insurance had lower odds of practice) ([23](#_ENREF_23)). Economic barriers mostly affected uptake of clinical breast examination and mammography ([11](#_ENREF_11), [57](#_ENREF_57)) and did not affect breast self-examination as some participants reported their preference for BSE because “*it is convenient as it doesn’t cost anything*” ([11](#_ENREF_11)).

**1b. Demographic factors**

Age was found to be a factor associated with screening uptake in several studies, though they differed between what age is associated with screening uptake as a barrier or facilitator. Some studies found uptake of BCS practices to increase with increasing age ([13](#_ENREF_13), [23](#_ENREF_23), [30](#_ENREF_30), [35](#_ENREF_35), [46](#_ENREF_46), [47](#_ENREF_47)). However, other studies reported that younger women had better odds of performing BCS ([38](#_ENREF_38), [51](#_ENREF_51), [66](#_ENREF_66)).

One good quality study reported rural women to have fewer odds (OR = 0.83, p < 0.001) of undergoing BCS ([23](#_ENREF_23)). This concurred with findings from a lower-quality study that noted urban participants to be more likely to perform BCS ([58](#_ENREF_58)). It was noted that rural women often lack social infrastructures such as health facilities in their vicinity and access to information such as through the media that would influence their participation in BCS ([23](#_ENREF_23), [58](#_ENREF_58)).

Several studies found a positive correlation between higher levels of education and uptake of BCS ([9](#_ENREF_9), [22](#_ENREF_22), [24](#_ENREF_24), [39](#_ENREF_39), [42](#_ENREF_42), [47](#_ENREF_47), [51-53](#_ENREF_51), [58](#_ENREF_58), [63](#_ENREF_63)). Conversely, women with primary level education and lower were less likely to be screened for BC than those with higher levels of education ([23](#_ENREF_23), [35](#_ENREF_35)). Not only were educated women more likely to undergo BCS but also women married to men with higher education status were also more likely to under BCS ([9](#_ENREF_9), [10](#_ENREF_10), [33](#_ENREF_33)).

**1c. Social factors**

Seven studies reported that having a family history of BC or familiarity with people with a history of BC is a facilitator to undergoing BCS, with odds of undergoing BCS being 1.7 (95%CI: 1.1, 2.6) to 7*:*14 (95% CI: 1.75, 25) ([10](#_ENREF_10), [24](#_ENREF_24), [31](#_ENREF_31), [32](#_ENREF_32), [36](#_ENREF_36), [38](#_ENREF_38), [40](#_ENREF_40), [42](#_ENREF_42), [43](#_ENREF_43), [49](#_ENREF_49), [51](#_ENREF_51), [52](#_ENREF_52), [56](#_ENREF_56)). Additionally, when participants were asked to give reasons on preference of undergoing particular screening interventions, studies reported that the commonest reasons for undergoing CBE and mammography were having a family history of BC ([9](#_ENREF_9)). One study however found that those with family history of BC were less likely to undergo BSE and CBE ([47](#_ENREF_47)). A study done on BSE also reported family pressure as one of the reasons for undertaking BSE ([38](#_ENREF_38)).

In addition, a total of six studies reported marital status as a factor related to BCS practice with not being married as a barrier to BCS uptake, with odds of screening among “not married” being 0.25-0.68 compared to the married ([23](#_ENREF_23), [34](#_ENREF_34), [47](#_ENREF_47), [48](#_ENREF_48), [53](#_ENREF_53)). Of note, poor social support has been shown as a barrier to undertaking BCS even among married women ([46](#_ENREF_46), [57](#_ENREF_57)).

Three of the included studies found a positive correlation with odds of practice being 3.85-5.51 (95%CI:3.45-8.79; 95%CI:1.82-8.33) among women who reported to have discussed BCS with others ([27](#_ENREF_27), [36](#_ENREF_36), [49](#_ENREF_49)). Similarly, participants who reported knowing someone screened for BC were more likely to undergo BCS (AOR=2.2; 95%CI: 1.10–4.38) ([24](#_ENREF_24)). Other social factors positively associated with BCS practice were: religion ([9](#_ENREF_9), [12](#_ENREF_12)), and long breast-feeding durations (13–24 months) ([33](#_ENREF_33)).

**1d. Perceptions and attitude**

Some of the major influencers of BCS uptake were patients’ perceptions and attitude on BC and BCS. Several studies discussed perceived confidence to undergo BCS (BSE specifically) and found that women who had a good perceived confidence to do BSE were more likely to undergo the BCS intervention ([8](#_ENREF_8), [10](#_ENREF_10), [34](#_ENREF_34), [37](#_ENREF_37)).

Studies in this review also showed that women who had good perceived susceptibility to develop BC were more likely to practice BCS as compared to women who had low perceived susceptibility ([8](#_ENREF_8), [35](#_ENREF_35), [37](#_ENREF_37), [40](#_ENREF_40), [46](#_ENREF_46), [54](#_ENREF_54), [62](#_ENREF_62)). Several studies reported that participants who did not engage in any form of BCS (BSE, CBE or RS) attributed their non-practice to be due to “*not being at risk of BC*” ([9](#_ENREF_9), [25](#_ENREF_25), [40](#_ENREF_40), [54](#_ENREF_54)).

Additionally, those with a favourable attitude towards BCS with perceived benefit were at higher odds of practicing BCS compared to those who did not perceive any benefit in undergoing BCS ([24](#_ENREF_24), [28](#_ENREF_28), [34](#_ENREF_34), [37](#_ENREF_37), [39](#_ENREF_39), [43](#_ENREF_43), [46](#_ENREF_46), [49](#_ENREF_49), [51](#_ENREF_51), [52](#_ENREF_52), [54](#_ENREF_54), [62](#_ENREF_62), [63](#_ENREF_63)). Moreover, among studies that reported practice of BCS by mammography, most participants did not perceive benefit of undergoing mammography and some perceived harm. Attitude towards mammography was generally negative, the main barrier being lack of information as most said that mammography has the potential of causing harm; “*I know I cannot go for it frequently as I fear getting cancer*” another said: “*I was told that machine presses your breasts so hard causing pain, so I better do BSE and go for CBE instead of going for those other things*” ([11](#_ENREF_11)).

Several studies reported that participants who did not engage in any form of BCS (BSE, CBE or RS) attributed their non-practice to be due to absence of any breast symptoms ([9](#_ENREF_9), [24](#_ENREF_24), [27](#_ENREF_27), [28](#_ENREF_28), [30](#_ENREF_30), [34](#_ENREF_34), [38](#_ENREF_38), [41](#_ENREF_41), [43](#_ENREF_43), [44](#_ENREF_44), [47](#_ENREF_47), [49-52](#_ENREF_49)). Also, another stated reason for not pursuing any form of BCS was fear of the results ([24](#_ENREF_24), [25](#_ENREF_25), [31](#_ENREF_31), [41](#_ENREF_41), [49](#_ENREF_49), [52](#_ENREF_52), [57](#_ENREF_57)). Urban women were more likely to report barriers related to fear ([57](#_ENREF_57)) compared to rural women. However, some participants in other studies reported to undergo BCS because they fear being diagnosed with advanced BC ([35](#_ENREF_35), [39](#_ENREF_39)).

Other related reasons that were stated to be barriers to BCS were negligence ([10](#_ENREF_10), [24](#_ENREF_24), [31](#_ENREF_31), [40](#_ENREF_40), [43](#_ENREF_43), [45](#_ENREF_45)) and forgetfulness ([24](#_ENREF_24), [25](#_ENREF_25), [30](#_ENREF_30), [31](#_ENREF_31), [41](#_ENREF_41), [43](#_ENREF_43), [45](#_ENREF_45), [51](#_ENREF_51), [52](#_ENREF_52), [55](#_ENREF_55)). In addition, studies done on BSE also stated that other participants reported BCS not to be important hence they did not pursue it ([27](#_ENREF_27), [34](#_ENREF_34), [54](#_ENREF_54)). Among studies done on CBE, participants also reported having busy schedules and laziness to go to the health facilities to be among the reasons they don’t pursue CBE ([25](#_ENREF_25), [55](#_ENREF_55)).

**1e. Knowledge and Awareness**

The most salient individual factor that facilitates an individual’s participation in BCS is the person’s knowledge about BC and BCS. The most prevalent reason for not undertaking any form of BCS was the lack of knowledge about the various forms of BCS ([24](#_ENREF_24), [57](#_ENREF_57)). Additionally, studies done on BSE showed that those who reported not to practice BSE reported not knowing how to perform BSE as the major barrier to performing screening ([10](#_ENREF_10), [28](#_ENREF_28), [30](#_ENREF_30), [31](#_ENREF_31), [34](#_ENREF_34), [38-40](#_ENREF_38), [43-45](#_ENREF_43), [50](#_ENREF_50), [54](#_ENREF_54), [55](#_ENREF_55)). Majority of the included studies investigated relationships between knowledge and awareness about BC and BCS to BCS practice and they all found a positive correlation ([9](#_ENREF_9), [10](#_ENREF_10), [13](#_ENREF_13), [24](#_ENREF_24), [26](#_ENREF_26), [28](#_ENREF_28), [30](#_ENREF_30), [31](#_ENREF_31), [33](#_ENREF_33), [34](#_ENREF_34), [36](#_ENREF_36), [37](#_ENREF_37), [39](#_ENREF_39), [40](#_ENREF_40), [43](#_ENREF_43), [49](#_ENREF_49), [51](#_ENREF_51), [52](#_ENREF_52), [55-57](#_ENREF_55), [62](#_ENREF_62), [63](#_ENREF_63)). Among female university students, students enrolled in clinical studies were more knowledgeable than their counterparts and hence more likely to practice BCS ([29](#_ENREF_29), [32](#_ENREF_32), [33](#_ENREF_33)).

Sources of information commonly mentioned were from the media (radio and television), friends health facility, lectures and the internet ([27](#_ENREF_27), [36](#_ENREF_36), [38](#_ENREF_38), [41](#_ENREF_41), [50](#_ENREF_50), [51](#_ENREF_51), [53](#_ENREF_53), [56](#_ENREF_56)). Source of information served as both a barrier and a facilitator for screening uptake. One study reported that participants who heard about BSE from media and friends were less likely to report correct knowledge and practice than those who got the information from the internet and health facilities ([53](#_ENREF_53)). In contrast, other studies showed those who got their information from the media and friends were more likely to practice BCS ([27](#_ENREF_27), [36](#_ENREF_36), [38](#_ENREF_38), [56](#_ENREF_56)).

1. **Provider (Health-system) related factors**

Among the included studies, one study found that those who were recommended by health professions were more likely to undertake BCS (AOR=5.0; 95%CI: 2.35–10.68)] ([24](#_ENREF_24)). Similarly, women who had practiced clinical breast examination were at increased odds of undergoing other methods of BCS compared to those who had not practiced CBE (AOR:2.69, 95%CI:1.31-5.52) ([27](#_ENREF_27), [66](#_ENREF_66)).

Those who reported to undertake clinical breast examination were less concerned about wasting a doctor’s time ([66](#_ENREF_66)). In contrast, those who reported not to prefer clinical breast examination reported factors such as wasting doctor’s time (if no breast complaint), long queues, poor quality of services and lack of information about provision of such services to be barriers to undertaking CBE ([25](#_ENREF_25)).

In addition, women who received regular care at health facilities were also more likely to have undergone a BCS intervention compared to those who did not attend health facilities ([55](#_ENREF_55), [58](#_ENREF_58)). This may have been related to receiving BC education as women who received previous BC education showed significantly higher downstaging practices ([58](#_ENREF_58)). Additionally, long distance to health facilities (incurring transport cost) was stated as a reason of not pursuing BCS (mainly CBE and mammography) ([25](#_ENREF_25), [51](#_ENREF_51), [56](#_ENREF_56), [66](#_ENREF_66)).
